# Minimally Invasive Repair of Pectus Excavatum: A Lifeline to Quality of Life

**DOI:** 10.3390/jcm13226888

**Published:** 2024-11-15

**Authors:** Mostafa Higaze, Mohamed Anwar Haj Khalaf, Chirag Parjiea, Horia Sirbu

**Affiliations:** 1 Division of Thoracic Surgery, Erlangen University Hospital, 91054 Erlangen, Germany; mohamed.hajkhalaf@uk-erlangen.de (M.A.H.K.);; 2 Faculty of Medicine, Friedrich-Alexander University Erlangen-Nürnberg (FAU), 91054 Erlangen, Germany

**Keywords:** pectus excavatum, MIRPE, quality of life

## Abstract

**Background**: Pectus excavatum, a deformity of the chest wall, can lead to significant emotional and social challenges, adversely affecting an individual’s overall well-being. While the Nuss procedure is a well-established treatment, this study evaluates the impact of a modified, minimally invasive approach on patients’ quality of life. **Methods**: This retrospective study analyzed patients who underwent minimally invasive pectus excavatum repair (MIRPE) from 1 January 2010 to 31 January 2024. Collected data included demographics, operative details, and patient outcomes. Health-related quality of life (HRQoL) was assessed using the SF-36 questionnaire, administered preoperatively and at least four weeks post-surgery. **Results**: Two hundred thirty-four patients (195 male, mean age 20.5 ± 6 years) underwent MIRPE. Postoperative results showed statistically significant improvements in all dimensions of HRQoL (Physical Component Summary Score (PCS) *p* = 0.007; Mental Component Summary Score (MCS) *p* < 0.001). Importantly, postoperative HRQoL scores were not just improved but comparable to those of the general German population, providing reassurance and confidence in the effectiveness of MIRPE. **Conclusions**: This study demonstrates that MIRPE significantly enhances patients’ quality of life (HRQoL). The improvements observed post-surgery bring HRQoL to levels akin to healthy individuals in the German population. These findings underscore the profound positive impact of MIRPE on patients’ well-being, offering hope and optimism for the future of pectus excavatum treatment.

## 1. Introduction

Pectus excavatum (PE) is the most common congenital chest wall deformity, characterized by an inward depression of the sternum. It is estimated to affect approximately 1 in 300–400 births [1,2]. The current hypothesis suggests that excessive growth of costal cartilage during pubertal development contributes to the pathogenesis of PE [2]. To quantify the severity of PE and guide surgical decision-making, established metrics such as the Haller Index (HI) and the Correction Index (CI) derived from computed tomography (CT) scans or magnetic resonance imaging (MRI) are used. Surgical intervention is usually considered when the HI ≥ 3.25 or the CI ≥ 28% [3,4]. Our goal in this study is to examine the dynamics of health-related quality of life (HRQoL) in patients after undergoing minimally invasive repair of pectus excavatum (MIRPE).

## 2. Materials and Methods

This retrospective analysis was conducted at the Department of Thoracic Surgery, University Hospital Erlangen, Germany. We reviewed the electronic medical records of 234 patients (195 males, 39 females; mean age, 20.5 ± 6 years) who underwent MIRPE between 1 January 2010 and 31 January 2024. Inclusion criteria were a Haller index (HI) ≥ 3.25, documented evidence of cardiac compression, cardiopulmonary limitations, or significant psychosocial sequelae. A single high-volume surgeon performed all surgical procedures. The mean preoperative HI was 4.36 ± 1.4. A detailed description of presenting symptomatology is provided in Table 1.

All patients completed a Short-Form-36 Health Survey questionnaire (SF-36) preoperatively and at least four weeks after surgery. Each patient was given an anonymized ID so the data could be documented and analyzed independently of the individuals. In this study, we investigated whether and how the HRQoL of PE patients is affected by MIRPE. To represent the level of subjective disability, the SF-36 questionnaire is used worldwide for clinical diagnostics and scientific studies. Using this questionnaire, the patient can self-report their quality of life without being influenced by their current health status or age. The patient is asked to select from the possible answers the one closest to their condition. The response categories vary from a dichotomous yes-no format to a six-item response scale [5,6]. The SF-36 includes two health indicators: “Functional status” and “Well-being”; these, in turn, are distributed across eight domains (see Table 2).

**Analyzing the HRQoL using SF-36.** This study evaluated the impact of MIRPE on the HRQoL in patients with PE. Preoperative and postoperative HRQoL scores were collected, compared, and analyzed using a validated instrument. Following MIRPE, a statistically significant improvement was observed in all eight HRQoL domains compared to preoperative scores. This improvement surpassed or equaled the German population norms [7]. These findings suggest that MIRPE can significantly improve HRQoL across various domains in PE patients, enhancing overall quality of life.

**Preoperative Assessment.** All patients underwent a comprehensive preoperative evaluation, which included a detailed physical examination, echocardiography, cardiopulmonary exercise testing, electrocardiography, and chest imaging using CT or MRI. The Haller index, a well-validated metric for measuring the severity of PE, was calculated for each patient, as Haller et al. [4] described. Surgical intervention was considered necessary based on established criteria, including a Haller index ≥ 3.2, documented evidence of cardiac compression, objective findings of cardiopulmonary limitations, significant or progressive symptoms, and a negative impact on psychosocial well-being. To assess the effects of the surgery, the SF-36 questionnaire was used. This questionnaire is a well-established and reliable tool for evaluating health-related quality of life (HRQoL). It comprises eight domains, each scored independently and subsequently summarized into two primary components: the Physical Component Summary (PCS) and the Mental Component Summary (MCS). These composite scores comprehensively represent a patient’s physical and mental health functioning (Table 2 and Table 3) [8].

**Statistical Analyses.** Statistical analyses were performed using IBM SPSS Statistics (Version 29.1, IBM Corp., Armonk, NY, USA). The normality of continuous variables was assessed using the Shapiro–Wilk test. Descriptive statistics are presented as mean ± standard deviation (SD) for normally distributed data and median and interquartile range (IQR) for skewed data. Independent-samples t-tests were used to compare normally distributed continuous variables preoperatively and postoperatively. Mann–Whitney U tests were employed for skewed data. Statistical significance was set at a *p*-value less than 0.05.

**Figure 1:**
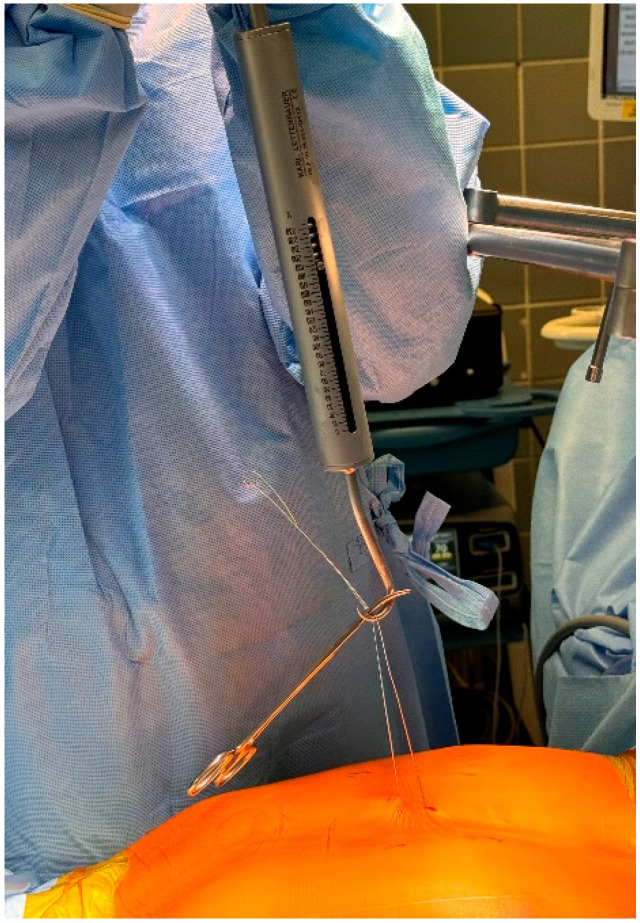
The tension needed to lift the sternum is measured using a Tensiometer.

**Figure 2:**
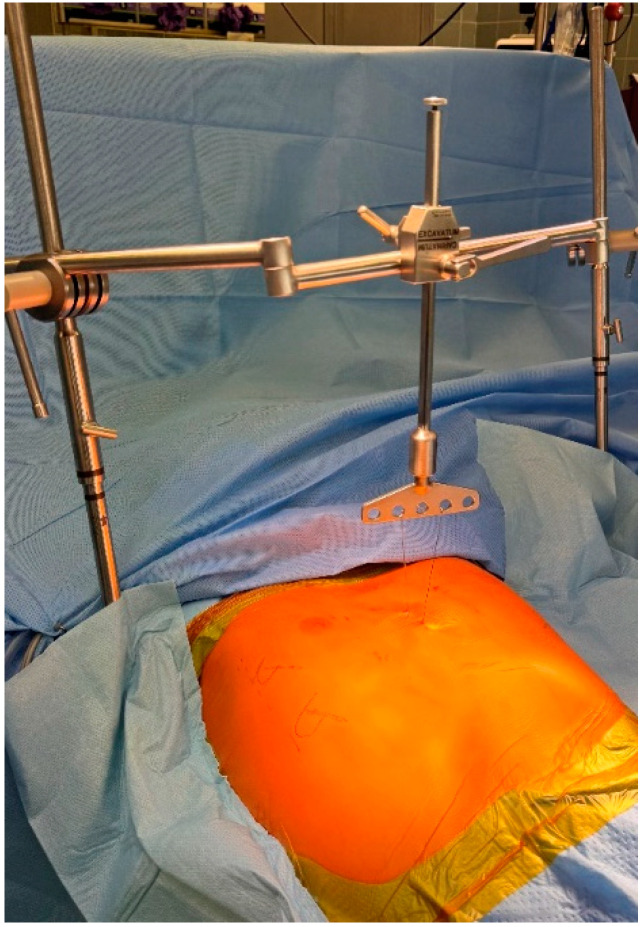
The “Osasun Track” is a sternum lifter designed by Ansabere Surgical specifically for intraoperative sternal elevation in cases of pectus excavatum.

**Surgical technique.** The Nuss procedure is a minimally invasive surgical technique primarily used to correct pectus excavatum in young children. Our team has continually improved this technique, developing a modified Nuss technique that has enhanced safety and effectiveness over the years. During the procedure under general anesthesia, the patient is positioned supine with slightly elevated arms. The deepest point is marked, and a steel wire is introduced transsternally. The wire was used for the tensiometric test to quantify the chest wall rigidity and for the sternal elevation maneuver (Figure 1). Bars were custom bent. We sized the bars so that the bar end extended only a maximum of 3 cm over the anterior axillary line. Small bilateral incisions were made between the anterior and mid-axillary lines, and submuscular pockets were developed. A Sternum lifter (Osasun Track—Co. Ansabere Surgical—Navarra, Spain) ensures precise and continuous sternal elevation (Figure 2), while a 5 mm thoracoscope provides meticulous visual guidance through a small incision on the right side. No carbon dioxide insufflation is necessary. A guiding instrument known as a Lorenz dissector is skillfully employed to facilitate the placement of bars, with a second bar positioned 1 or 2 interspaces below the upper bar. Continuous sternal elevation minimizes rotation forces throughout this process as the bars are meticulously rotated and fixed. After the bars are securely fixed to the ribs, they remain in place for three years before a subsequent surgical procedure is performed to safely remove them.

**Postoperative Care.** Chest radiography was performed immediately after surgery to confirm bar placement and exclude pneumothorax or hemothorax. Patient-controlled intravenous analgesia (PCIA) was used for pain management during the first 48 h. Physiotherapy was initiated on postoperative day one for all patients. Discharge criteria included adequate patient mobility and effective pain control. Standardized follow-up visits were scheduled at two weeks, six months, and annually postoperatively.

**Complications.** MIRPE is generally considered a safe procedure with a well-defined complication profile and low overall incidence [9,10]. Potential complications associated with MIRPE include a bar flipping or sliding, pneumothorax or hemothorax requiring intervention, wound infection, pleural effusion, and pericarditis or cardiac injury [9,11]. There were no mortalities or cases of thoracic outlet syndrome, pericarditis, cardiac injury, or sternal injury. Table 4 presents a comprehensive overview of all complications.

## 3. Results

Clinical data from 234 PE patients (83.5% male) who underwent MIRPE between 2010 and 2024 were retrospectively collected and analyzed. The age of the patients was 21.2 ± 6.61 years. The HI was 4.34 ± 1.48. A good postoperative outcome was achieved with one bar in 45.7%, two bars in 51.2%, and three bars in 3% of the patients. The number of implanted bars was selected based on intraoperative tensiometric data. The hospital stay was 7.7 ± 4.5 days, and the bars were removed after 4.7 ± 2.96 years (see Table 4).

**Figure 3:**
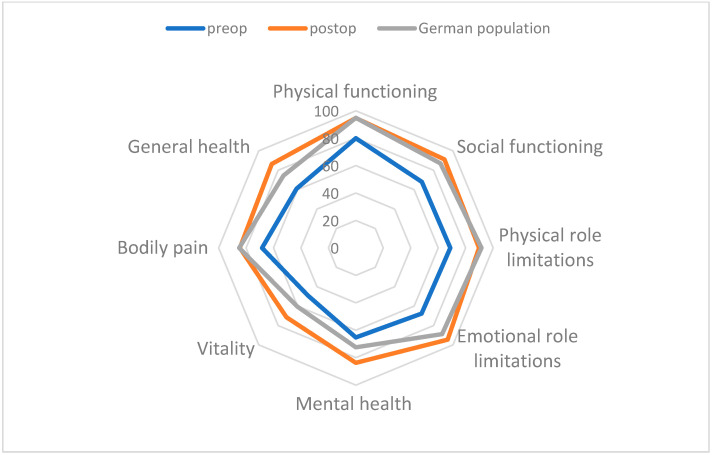
This radar chart visualizes changes in eight health-related categories before and after MIRPE, compared to the general population.

Our study observed notable enhancements in patients’ quality of life following surgical treatment. There was a significant improvement in physical functioning, evidenced by an increased ability to carry out daily activities (PF: 80.02 to 94.68; *p* = 0.087). Likewise, limitations imposed by the underlying condition on daily life decreased significantly (PRL: 68.78 to 89.69; *p* = 0.016). Pain scores also improved considerably statistically (PA: 68.34 to 84.50; *p* = 0.002). Patients subjectively reported better overall health (GH: 61.03 to 86.54; *p* < 0.001) and higher energy levels (VI: 49.23 to 71.43; *p* < 0.001). Social interactions improved significantly (SF: 68.05 to 91.27; *p* < 0.001). Moreover, the surgery had a positive impact on emotional well-being (ERL: 67.72 to 94.53; *p*= 0.003) and overall mental health (MH: 65.19 to 83.75; *p* < 0.001) (see Figure 3 and Table 5).

## 4. Discussion

The World Health Organization defines “quality of life” as an individual’s perception of their life in the context of culture and values [12]. Patients with pectus excavatum (PE) often experience psychological effects, avoiding sports activities and feeling uncomfortable with their appearance. Surgical correction has been shown to improve physical and psychosocial function, posture, and emotional difficulties and increase social awareness [13]. It also improves cardiac and pulmonary function in PE patients [14,15,16,17].

The MIRPE can lead to varying quality of life outcomes. Age, gender, deformity severity, and existing health conditions can influence post-surgery improvement [10]. Surgical complications like bar displacement or infection can negatively impact patient satisfaction and recovery [18]. Patients with severe deformities may experience longer recovery and lower satisfaction with cosmetic results. However, complications are rare, and patients generally experience improved satisfaction.

Our study showed significant findings. Every dimension of HRQoL saw noticeable improvement after MIRPE. We also compared our preoperative mean values with Germany’s adult health survey—ours were lower than the normative data. In comparison, the mean values measured postoperatively were like those of the norm data, and in the domains “GH, “VI, and “MH”, they were even higher. Patients expressed high satisfaction with their self-image and quality of life. Based on the values collected and analyzed independently from the patient, MIRPE significantly increases HRQoL. The impact of the reduced physiological function is not fully understood; however, it may explain the severity of physical limitations reported by patients. O’Keefe et al. (2013) investigated the cardiopulmonary effects of PE correction. Improvement in self-perceived well-being and appearance was found to have a more significant impact than improvement in pulmonary function and fitness [13]. After MIRPE, our patients reported substantial enhancements in their physical functioning (PF: 80.02 vs. 94.68) and physical role function (PRL: 67.78 vs. 89.69). Steinmann et al. (2011) reported comparable results; the body image was disturbed in PE patients compared to the norm sample [19].

The social activities of PE patients were disrupted. Compared to the control group, PE patients had a reduced social functioning ability (SF 68.05 vs. 87.1). PE patients experienced much lower ‘Emotional Role Limitation’ than the control group (ERL: 67.72 vs. 88.8). After surgery, our patients reported improvements in emotional well-being and self-esteem, as well as better physical and social activities, resulting in an elevated HRQoL. This highlights the positive effects that MIRPE can have. The psychosocial impact of PE is significant. After MIRPE, we observed a notable improvement in the patient’s ability to participate in daily life. Vitality (VI 49.23 vs. 71.43) and mental health (MH 65.19 vs. 83.75) recovered significantly.

Each dimension’s examination revealed significant improvements in patients’ psychosocial and physical health after MIRPE. Our postoperative outcomes were notably aligned with the German norm. Our data indicated that MIRPE enhances patients’ quality of life. There was a significant boost in psychosocial and physical function. The postoperative questionnaires, completed between 4 weeks and 10 years post-surgery, confirm that MIRPE’s positive effects on physical and psychosocial functioning are long-lasting.

This study’s findings offer crucial insights into the effect of minimally invasive repair of pectus excavatum on patients’ quality of life. Through a retrospective analysis, the study highlights the significant impact of MIRPE on patients’ HRQoL. By thoroughly reviewing the electronic medical records of 234 patients who underwent MIRPE, researchers noted substantial improvements in various HRQoL dimensions post-surgery. These improvements were statistically significant, as reflected in the Physical and Mental Component Summary Scores (PCS and MCS), with *p*-values of 0.007 and <0.001, respectively. Notably, postoperative HRQoL scores almost matched those of the healthy German population, demonstrating MIRPE’s transformative effect on patient well-being. This normalization to healthy levels emphasizes MIRPE’s success in improving both physical conditions and emotional and social functioning.

These findings support earlier research showing surgical intervention benefits for QoL [10], including MIRPE. However, this study delves into MIRPE’s specific impact on HRQoL, offering valuable insights for clinicians and patients. Although short-term studies yield promising results, longitudinal studies evaluating HRQoL outcomes years after surgery are limited. Future research should focus on long-term follow-ups to assess the durability of HRQoL improvements and discover any late-developing complications or declines in outcomes. In summary, the MIRPE significantly enhances HRQoL for those affected. Even though studies repeatedly show positive QoL outcomes post-surgery, individual differences and the risk of surgical complications highlight the need for personalized patient-centered care and continued research to optimize results, ensuring the best possible QoL for patients.

The findings of this study are promising; however, several limitations need to be acknowledged. Firstly, the retrospective nature introduces biases typical of such studies, including selection bias and the possibility of incomplete or inaccurate data collection. Additionally, the reliance on electronic medical records may need more data depth and granularity. The gender distribution was mainly male, potentially affecting the detection of significant gender differences. Before the correction, patients expressed concerns about cosmetic deformities and experienced considerable body image issues. Body image was not measured separately but was included in the self-esteem scale.

In summary, the questionnaires used are well-designed to evaluate disease-specific HRQoL changes. The findings verify that MIRPE improves patients’ psychosocial and physical functions consistently when compared to the German norm and underscore MIRPE’s notable impact on patients’ HRQoL, demonstrating its vital role in enhancing the quality of life for those with pectus excavatum. By addressing the condition’s physical, emotional, and social aspects, MIRPE is highlighted as a promising treatment option that merits additional clinical investigation and consideration. Future prospective studies with larger groups and longer follow-up periods are suggested to substantiate further and broaden these findings.

## Figures and Tables

**Table 1:** Baseline patients’ characteristics.

Characteristics	
Age, y, median (IQR; range)	18.7 (6.2; 14–51)
Gender, *n* (%)	
Male	195 (83)
Female	39 (17)
Haller index, median (IQR)	4.0 (1.3)
Preoperative symptoms, n (%)	
Exercise intolerance	199 (85.0)
Psychosocial complaints	156 (66.7)
Dyspnea at rest	168 (71.8)
Cardiovascular Symptoms	134 (57.3)
Chest pain other than angina pectoris	80 (34.2)
Back pain	83 (35.5)
Muscle stiffness	38 (16.2)
Gastrointestinal Symptoms	8 (3.4)

IQR, interquartile range.

**Table 2:** SF-36, the health indicators, and the eight domains.

	Dimension (abbr.)	No° of Questions
Functional status	Physical functioning (PF)	10
	Social functioning (SF)	2
	Physical role limitations (PRL)	4
	Emotional role limitations (ERL)	3
Well-being	Mental health (MH)	5
	Vitality (VI)	4
	Bodily Pain (PA)	2
	General health perception (GH)	5
	Health change	1
	Total	36

**Table 3:** Description of SF-36 domains.

Physical Functioning (PF)	This domain assesses the patient’s ability to perform basic and advanced physical activities of daily living.
Social Functioning (SF)	This domain evaluates the patient’s ability to participate in social activities and maintain social relationships.
Physical role limitations (PRL)	This domain evaluates the impact of physical health on the patient’s ability to carry out work or other significant role-related activities.
Emotional role limitations (ERL)	This domain assesses the impact of emotional problems on the patient’s ability to perform work or other role-related activities.
Mental health (MH)	This domain reflects the patient’s overall emotional and psychological well-being
Vitality (VI)	This domain assesses the patient’s energy levels and overall sense of well-being.
Bodily pain (PA)	This domain measures the intensity and interference of pain on daily life.
General health (GH)	This domain captures the patient’s subjective assessment of their overall health status.

**Table 4:** Outcomes.

Characteristics	
Bars inserted, n (%)	
1 bar	86 (37)
2 bars	140 (60)
3 bars	8 (3)
Bar length, inches, median (IQR)	
Cranial bar	12 (2)
Caudal bar	12 (1)
Tensiometrie, Nm, median (IQR)	160 (50)
Operation time, min, median (IQR)	89 (38)
Length of hospital stay, d, median (IQR)	7 (2)
Postoperative morbidity, n (%)	
Bar displacement requiring reoperation	9 (3.8)
Empyema	2 (0.85)
Deep wound infection requiring reoperation	3 (1.28)
Pleural effusion requiring intervention	2 (0.85)
Pneumothorax requiring intervention	10 (4.27)
Hemothorax requiring intervention	2 (0.85)
Recurrence after bar removal	2 (0.85)
Wound infection	4 (1.7)
Chronic pain	5 (2.13)

*p* < 0.050 is considered statistically significant; IQR, interquartile range.

**Table 5:** Pre- and postoperative values of all eight domains in SF-36.

Characteristics	German Population	Before Surgery	After Surgery	*p*-Value
Physical functioning (PF)	94.9 (94.0–95.7)	80.02 ± 20.7	94.68 ± 14.0	0.087
Social Functioning (SF)	87.1 (85.6–88.7)	68.05 ± 28.8	91.27 ± 16.2	<0.001
Physical role limitations (PRL)	91.5 (90.4–92.6)	68.78 ± 38.7	89.69 ± 27.3	0.016
Emotional role limitations (ERL)	88.8 (87.4–90.1)	67.72 ± 40.3	94.53 ± 19.7	0.003
Mental health (MH)	72.4 (71.2–73.5)	65.19 ± 19.8	83.75 ± 13.6	<0.001
Vitality (VI)	60.4 (59.4–61.4)	49.23 ± 19.6	71.43 ± 19.0	<0.001
Bodily pain (PA)	85.0 (83.5–86.5)	68.34 ± 27.7	84.50 ± 21.7	0.002
General health (GH)	74.5 (73.3–75.6)	61.03 ± 23.2	86.54 ± 16.8	<0.001
Summary				
Physical Component Summary Score (PCS)	55.8 (55.4–56.2)	47.36 ± 10.2	53.99 ± 7.6	0.007
Mental Component Summary Score (MCS)	48.0 (47.3–48.7)	43.70 ± 11.5	54.42 ± 7.3	<0.001

*p* < 0.050 is considered statistically significant; IQR, interquartile range.

## Data Availability

The data presented in this study are available on reasonable request from the corresponding author.
